# Temperature-Dependent Anisotropic Refractive Index in β-Ga_2_O_3_: Application in Interferometric Thermometers

**DOI:** 10.3390/nano13061126

**Published:** 2023-03-21

**Authors:** Daniel Carrasco, Eva Nieto-Pinero, Manuel Alonso-Orts, Rosalía Serna, Jose M. San Juan, María L. Nó, Jani Jesenovec, John S. McCloy, Emilio Nogales, Bianchi Méndez

**Affiliations:** 1 Department Materials Physics, Faculty of Physics, Complutense University of Madrid, 28040 Madrid, Spain; daniecar@ucm.es (D.C.); manalo01@ucm.es (M.A.-O.); bianchi@fis.ucm.es (B.M.); 2 Laser Processing Group, Instituto de Óptica (IO, CSIC), Serrano 121, 28006 Madrid, Spain; eva.nieto@csic.es (E.N.-P.); rosalia.serna@csic.es (R.S.); 3 Institute of Solid State Physics, University of Bremen, Otto-Hahn-Allee 1, 28359 Bremen, Germany; 4 Department de Física, Facultad de Ciencia y Tecnología, Universidad del País Vasco UPV/EHU, Apdo. 644, 48080 Bilbao, Spain; jose.sanjuan@ehu.eus (J.M.S.J.); maria.no@ehu.es (M.L.N.); 5 Crystals and Semiconductors Group, Institute of Materials Research, Washington State University, Pullman, WA 99164, USA; jani.jesenovec@baesystems.com (J.J.); john.mccloy@wsu.edu (J.S.M.)

**Keywords:** gallium oxide, nanowire, optical microcavity, thermometer, refractive index, FDTD, ellipsometry, photoluminescence

## Abstract

An accurate knowledge of the optical properties of β-Ga_2_O_3_ is key to developing the full potential of this oxide for photonics applications. In particular, the dependence of these properties on temperature is still being studied. Optical micro- and nanocavities are promising for a wide range of applications. They can be created within microwires and nanowires via distributed Bragg reflectors (DBR), i.e., periodic patterns of the refractive index in dielectric materials, acting as tunable mirrors. In this work, the effect of temperature on the anisotropic refractive index of β-Ga_2_O_3_ *n*(*λ*,*T*) was analyzed with ellipsometry in a bulk crystal, and temperature-dependent dispersion relations were obtained, with them being fitted to Sellmeier formalism in the visible range. Micro-photoluminescence (μ-PL) spectroscopy of microcavities that developed within Cr-doped β-Ga_2_O_3_ nanowires shows the characteristic thermal shift of red–infrared Fabry–Perot optical resonances when excited with different laser powers. The origin of this shift is mainly related to the variation in the temperature of the refractive index. A comparison of these two experimental results was performed by finite-difference time-domain (FDTD) simulations, considering the exact morphology of the wires and the temperature-dependent, anisotropic refractive index. The shifts caused by temperature variations observed by μ-PL are similar, though slightly larger than those obtained with FDTD when implementing the *n*(*λ*,*T*) obtained with ellipsometry. The thermo-optic coefficient was calculated.

## 1. Introduction

Gallium oxide in its monoclinic phase, β-Ga_2_O_3_, is the most stable among the different polytypes of this oxide and has been increasingly studied during the last six decades. It presents exceptional optical and electronic properties including an ultra-wide bandgap (4.9 eV) and a very high critical electric field, as well as very high thermal and chemical stability and radiation resistance [1]. Due to all of these properties, it is considered one of the most promising semiconductors for high power devices [2]. In addition, β-Ga_2_O_3_ (from now on, this phase will be indicated as Ga_2_O_3_) offers strong potential in photonics applications. For example, Ga_2_O_3_ thin films have been proposed in solar-blind ultraviolet (UV) photodetectors for fire/flame detection [3], while tunable luminescent devices based on bulk [4,5] or nanocrystalline Ga_2_O_3_ [6] have also been proposed. As a wide bandgap material, Ga_2_O_3_ shows tunable luminescence when suitably doped with optically active impurities.

Semiconducting quasi-1 dimensional (1D) micro- and nanowires (μW and nW) allow the miniaturization and optimization of several photonic devices because their optical properties can be controlled by modifying the material and/or by patterning artificial optical structures, among which optical microcavities are key elements. One type of photonic structure used to create optical microcavities is the distributed Bragg reflector (DBR): a structure where the refractive index varies periodically in space along a specific direction. This results in forbidden frequency bands for light propagation, also called stopbands. Electromagnetic waves with these frequencies cannot propagate along the axis perpendicular to the DBR interfaces; hence, they are mostly reflected. In a μW or nW that emits a luminescence band, a couple of correctly designed DBRs that reflect such a band along the wire axis result in the effective spatial confinement of the band due to a combination of total internal reflection, i.e., waveguiding, and reflections in the DBRs. This gives rise to resonance frequencies that can be analyzed as longitudinal, Fabry–Perot (F-P).

Recently, we proposed optical microcavities based on distributed Bragg reflector (DBR) patterning created by focused ion beam (FIB) lithography in Cr-doped Ga_2_O_3_ μW or nW, working in the near-infrared (near-IR) or near-UV ranges [7,8]. Furthermore, we demonstrated the application of such cavities as micrometer dimensioned, wide dynamical range thermometers [9]. Part of this application is based on the temperature dependence of the Ga_2_O_3_ anisotropic refractive index.

Progress on the applications of Ga_2_O_3_ in photonic devices requires the knowledge of the dispersion relations, i.e., wavelength-dependent refractive index, as a function of temperature, *n(λ*,*T)*. Furthermore, the inherent anisotropy of the monoclinic Ga_2_O_3_ phase is also translated into unique light–matter interaction in this oxide; hence, a rigorous study of dispersion relations in Ga_2_O_3_ should incorporate the anisotropic effects. A full study of *n*(λ,*T*) in this oxide still needs to be further discussed. Indeed, even though it is of great importance for the application of this very relevant material in photonics, just a few studies have been reported [9,10,11,12]. The work by Bhaumik et al. [10] used the prism method to obtain the refractive index at temperatures between room temperature (RT, 25 °C, i.e., 298 K) and 200 °C (473 K). However, the characterization was only performed for the refractive index along two crystal directions, i.e., [010] and perp.(100). On the other hand, Sturm et al. [11] used ellipsometry, and the analysis is similar to the one presented in this work. Nonetheless, there is a main difference because they studied the variation in the refractive index at low temperatures—from 10 K to 300 K (room temperature)—while we report data in the 295 K to 595 K range. It is not straightforward that the evolution of the refractive index at low temperatures can be extrapolated to higher temperatures.

In this paper, we fully analyze the refractive index temperature dependence in the three main crystal axes with the assistance of variable angle spectroscopic ellipsometry. Using that temperature dependence, complete simulations are performed, showing their accuracy by comparing them with the experimental μ-PL results in an actual microcavity where local temperature is varied by changing the excitation power of the laser.

## 2. Materials and Methods

Unintentionally doped (UID) Ga_2_O_3_ bulk crystal and Cr-doped Ga_2_O_3_ nW, both with (100) surfaces, were used to assess the anisotropic refractive index of Ga_2_O_3_ and optical resonances as a function of the temperature. All materials exhibit the monoclinic β-phase [9,13]. Bulk unintentionally doped (UID) crystals were grown by the Czochralski and vertical gradient freeze techniques, as recently summarized [13]. Cr-doped Ga_2_O_3_ nW were obtained by controlled thermally treated metal Ga at 1500 °C for 15 h under atmosphere conditions [7]. Selected nW were placed on a TEM grid and subsequently patterned with a FIB, FEI Helios NanoLab 650, in order to produce the designed DBRs that act as optical mirrors in an F-P cavity scheme, as described elsewhere [7,9]. In this way, an optical cavity with length in the range of micrometers successfully confines optical modes in the red range (690–750 nm), suitable for the Cr^3+^ intraionic emissions.

FDTD simulations were performed with the commercial OptiFDTD software, by Optiwave, to calculate the reflectivity of the DBRs, as well as the wavelength of the optical resonances resulting from the optical cavities at different temperatures using different models of *n(λ*,*T)*.

The morphology of the optical cavities based on Ga_2_O_3_-microwires was assessed by scanning electron microscopy (SEM) in an FEI Inspec instrument. Micro-photoluminescence (µPL) was carried out in a Horiba Jobin Yvon LabRAM HR800 confocal microscope, with an HeCd 325 nm or a HeNe 632.8 nm lasers as excitation sources. The excitation power was selected by using filters with different optical densities, OD 0.3, 0.6, 1 or 2. The aims of the experiments in the confocal microscope were twofold: (i) to assess the optical F-P resonances and (ii) to use the laser as a local heat source to increase sample temperature.

**Figure 1:**
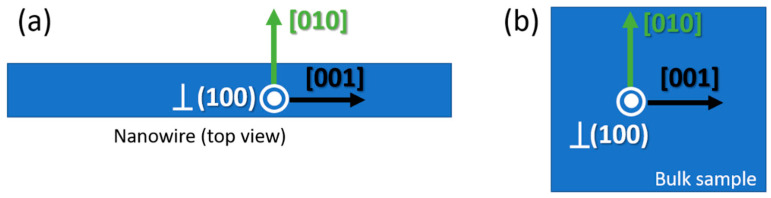
Sketch of the orientations of the crystal and the reference system, for both (**a**) nanowires and (**b**) bulk Ga_2_O_3_ crystals.

The ellipsometry measurements were carried out with a Woollam VASE ellipsometer on bulk Ga_2_O_3_ (100)-oriented crystals placed onto a hot plate that allows controlled sample heating and cooling (INSTEC equipment). The crystal was glued to the surface of the hot plate with silver paint in order to optimize the heat transfer. The ellipsometry data were acquired and analyzed with the WVASE software using the Mueller matrix formalism as described in the work by Schubert et al. [14]. This approach is necessary due to the anisotropic nature of the β-Ga_2_O_3_ crystal and allows the optical sample properties at a given angle of incidence and sample orientation to be obtained. In this approach, the measured data must be analyzed through a best-match model calculation procedure. It should be noted that spectroscopic ellipsometry is an indirect method and requires detailed model analysis procedures in order to extract relevant physical parameters. In this work, the fitting was performed using input data acquired in two different orientations perpendicular to each other. The orientations were defined using proper Euler angles with the ZXZ convention. The orientations used correspond to Euler angles (0, 90, 90) and (90, 90, 90), as shown in Figure S1 in Supplementary Materials. As light incidence angles, we used 60°, 65° and 70°. The wavelength range of the measurement was from 600 nm to 1200 nm. Figure 1 shows a sketch of the orientations of the crystal and the reference system, for both microwires and bulk crystals.

## 3. Results and Discussion

### 3.1. Temperature Dependence of the Refractive Index

**Figure 2:**
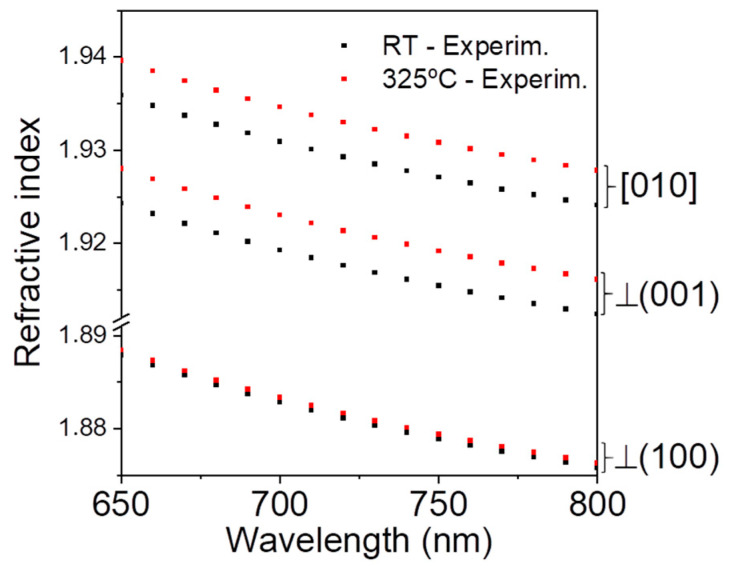
Refractive index values for the three axes at 25 °C (298 K) and 325 °C (598 K) obtained from ellipsometry data from Ga_2_O_3_ bulk crystal.

In order to obtain the temperature dependence of the dispersion relations in Ga_2_O_3_ in the visible range, where there is no absorption, ellipsometry analysis in a bulk (100) crystal at different temperatures was carried out. To analyze the ellipsometric data, we used the Mueller matrix formalism that enables the simultaneous fit of all the data measured at different angles and orientations [11,14,15]. Sturm et al. calculated the dielectric function (DF) tensor both at room temperature (RT) [15] and considering its temperature dependence in the range from 10 K to RT [11], where they reported a non-zero value for one of the non-diagonal terms. In the present work, dispersion relations were represented with Sellmeier formalism, considering the anisotropic nature of the crystal. This formalism was chosen because it is the one used in the OptiFDTD software. Figure 2 shows the refractive index values of the bulk sample derived from the ellipsometry measurements at room temperature, RT (298 K) and at 598 K. As it can be seen, a clear increase in the refractive indices when increasing T is observed for n_(010)_ and n_(001)_, while a much lower increment is obtained for n_(100)_. The results are in agreement with previous works that used Mueller matrix formalism [11,14,15]. It is worth mentioning that slight variations were obtained in the different works at room temperature, showing uncertainty in the quantitative evaluation of the dielectric function.

**Figure 3:**
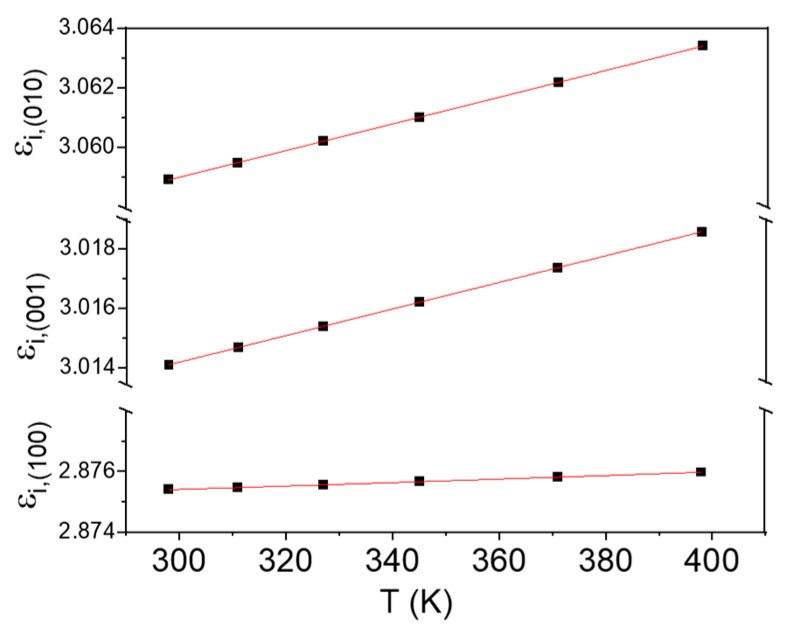
Anisotropic permittivity as a function of the temperature in the RT—400 K range.

The experimental points displayed in Figure 2 were fitted to the Sellmeier equation at each temperature, according to:

(1) $$n_{j}^{2}(\lambda ,T)=\varepsilon_{1,j}(T)+\frac{A\cdot \lambda^{2}}{\lambda^{2}-\lambda_{i}^{2}}$$

where *A* and *λ_i_* are Sellmeier parameters, which we let be the same for the three directions and be temperature-independent; *n_j_*(λ,T) is the temperature-dependent (*T*-dependent) dispersion relation for each main crystal direction, i.e., *j* = (100), (010), (001); finally, *ε_i_*_,*j*_(*T*) is the *T*-dependent static dielectric permittivity for direction *j*. These different, anisotropic values are used for FDTD simulations of both individual DBR reflectivity and light resonances in the Ga_2_O_3_ optical cavity, as described below. The *ε_i_*_,*j*_ (*T*) curves, as obtained from the fit of the ellipsometry data to the Sellmeier equation in the 300–400 K temperature range, are displayed in Figure 3. *ε_i_*_,*j*_(*T*) show good fits to quadratic expressions (solid lines), in agreement with the dependence reported for other semiconducting materials [16]. Table 1 shows the explicit expressions of *ε_i_*_,*j*_(*T*) obtained from these fits.

### 3.2. Fabry–Perot Resonances in Ga_2_O_3_ Optical Cavities

**Figure 4:**
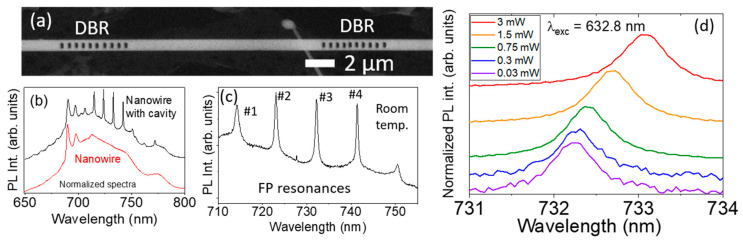
(**a**) SEM image of the microcavity patterned in a Cr-doped Ga_2_O_3_ nanowire, with the two DBRs indicated. (**b**) Comparison between RT μ-PL spectra from an as-grown nanowire and the microcavity shown in (**a**). Spectra were normalized and vertically shifted for the sake of clarity. (**c**) Blow up from (**b**) of the four main F-P resonance peaks observed in the microcavity, overlapping the broad phonon-assisted band in the near-IR range. Their positions are 714.2 nm, 723.0 nm, 732.1 nm and 741.3 nm and were labeled #1, #2, #3 and #4, respectively. (**d**) Detail of the evolution of the #3 resonant peak when changing the excitation power.

The previous results of the anisotropic *n_j_*(*λ*,*T*) are valuable in the analysis of photonic devices. Optical resonances in F-P cavities based on DBR mirrors built in a Cr-doped Ga_2_O_3_ nW, as described in the Experimental section, were analyzed. Figure 4a shows the SEM image of one analyzed microcavity. The cavity length between the DBRs is L = 13.0+/−0.1 μm, as it results from the SEM measurements. The comparison between room temperature (RT) μ-PL spectra from an as-grown nW and that of the microcavity are shown in Figure 4b. Excitation was obtained with the UV laser and an OD2 filter at RT. The spectral features of Cr^3+^-related intraionic transitions in the Ga_2_O_3_ host were observed: the sharp R lines at 689.8 and 696.6 nm (^2^E–^4^A_2_) as well as the phonon-assisted, broad band (^4^T_2_–^4^A_2_) [9,17,18]. Moreover, in the case of the microcavity, on top of the broad luminescence band, several sharp lines were observed, which were due to the F-P resonances caused by the spatial confinement of light. The detail of these resonances is shown in Figure 4c, where four main F-P peaks are well-defined at 714.2 nm, 723.0 nm, 732.1 nm and 741.3 nm. They were labeled #1, #2, #3 and #4, respectively. These peaks were previously shown to be of great interest for thermal sensing in a wide dynamic range (150–500 K) due to the nearly linear spectral redshift that they experience when the local temperature of the microcavity is increased. Furthermore, by μ-PL from one of these optical cavities introduced first in a cryostat and later in a heater, the calibration curves for the shift of these peaks were obtained between 150 K and 400 K for this sensing aim. The dependence of the peak positions with respect to temperature were nearly linear and are quantitatively expressed by the following expressions:

(2a) $$\lambda_{\#2}(T)=\lambda_{\#2}(295\;K)+1.15\cdot 10^{-5}T^{2}+5.5\cdot 10^{-3}T-2.65$$

(2b) $$\lambda_{\#3}(T)=\lambda_{\#3}(295\;K)+1.14\cdot 10^{-5}T^{2}+6.1\cdot 10^{-3}T-2.8$$

(2c) $$\lambda_{\#4}(T)=\lambda_{\#4}(295\;K)+1.19\cdot 10^{-5}T^{2}+6.2\cdot 10^{-3}T-2.91$$

where *λ* is expressed in nm and *T* in K. [9].

Figure 4d shows detailed μ-PL spectra of peak #3 when the microcavity was excited with the red laser at different powers, showing a redshift as excitation power increased. The resonant peaks of the PL spectra were fitted to Lorentzian functions to obtain their position. Figure S4 in the Supplementary Materials shows the excellent fit with the Lorentzian curves of the experimental PL peaks at three excitation powers for peaks #3 and #4, with an uncertainty of the peak position Δλ = 0.002 nm. The center and the width of the Lorentzian peaks are shown in Table 2. The shift is a consequence of the local heating of the microcavity as the laser power increases, raising its local temperature from 299 K up to 360 K, as calculated from Equation (2b). These data were later used to compare the position of the experimental F-P resonances at different temperatures with those obtained by FDTD simulations.

The physical mechanism for the redshift of the F-P resonances is mainly the variation in the refractive index, *n*(*T*), and, to a lesser extent, thermal expansion of the microcavity with temperature, as described by the following equation:

(3) $$\frac{d\lambda_{m}}{dT}=\lambda_{m}\left[{\left.\frac{1}{n_{\lambda_{m}}}\frac{dn}{dT}\right|}_{\lambda_{m}}+\frac{1}{L}\frac{dL}{dT}\right]=\lambda_{m}\left(\delta +\alpha \right)$$

where (1/*n_λm_*)·*dn*/*dT*|*_λm_* = *δ* is the thermo-optic coefficient—which in general depends on *λ_m_* and *T*—and (1/L)·*dL*/*dT* = *α* is the thermal expansion coefficient of the material, which has been considered to have the value 3.1 × 10^−6^ K^−1^ [19]. The thermal expansion can be considered as linearly dependent on the temperature, i.e., L_eff_(T) = L_eff_ (295 K) + *α* (T − 295). However, the thermo-optic coefficient in Ga_2_O_3_ has not been extensively studied so far. A value of δ ≈ 3.6–3.7 × 10^−5^ K^−1^ for the [010] and perp.(100) crystal directions was reported from prism coupling measurements [10], while an average value of δ ≈ 1.77 × 10^−5^ K^−1^ was calculated from the interferometry thermometry in [9]. These results are further discussed below, in the light of the FDTD simulations and experimental results obtained in this work.

### 3.3. FDTD Simulation of the Temperature-Dependent F-P Resonances’ Positions

**Figure 5:**
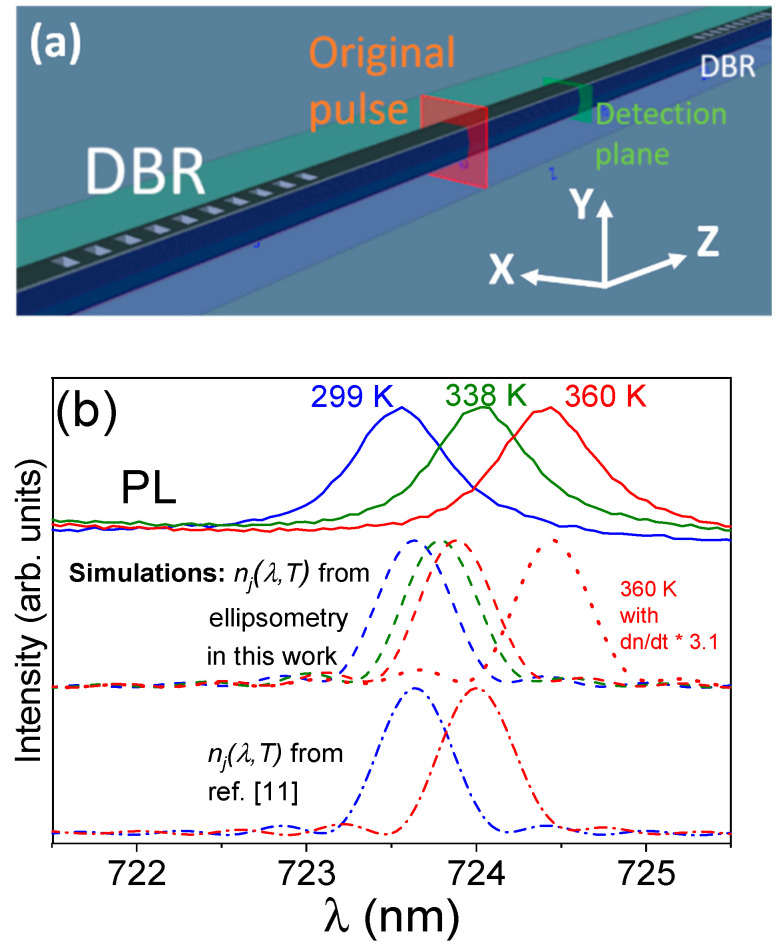
(**a**) Simulation procedure, with the schematic of the OptiFDTD Designer module. The red plane is the pulse source, while the green plane is the Poynting vector detection plane. The defined axes are shown in the lower, right corner. (**b**) Comparison of normalized experimental μ-PL spectra of peak #2 at three different temperatures (solid lines) with simulations at such temperatures, using both the anisotropic, temperature-dependent refractive index calculated from ellipsometry in this work (dashed lines) and that calculated from the data by Sturm et al. [11]. Dotted line shows the resonance at 360 K by using *n_j_*(*λ*,*T*) obtained when multiplying by a 3.1 factor the dn/dT value calculated by ellipsometry in this work.

The achievement of reliable T-dependent dispersion relations, obtained by ellipsometry and using the Mueller matrix formalism, for the diagonal elements of the dielectric tensor, allows us to further analyse the optical resonances in the F-P cavity built on Ga_2_O_3_ microwires. The temperature dependence of the μ-PL resonances (F-P peaks) were simulated by FDTD simulations, taking into account the anisotropic character of the refractive index in Ga_2_O_3_ along the principal axes and the particular crystallographic directions in the studied microcavity. This is schematically shown in Figure 5a, where assignment of the crystal directions (see Figure 1a) is X (perpendicular to nW axis and to optical axis) corresponding to crystal direction [010], Y (parallel to optical axis) to the perp. (100) planes’ direction and Z (along nW axis) to the perp.(001) planes’ direction. Finally, Sellmeier parameters are calculated from the data in Table 1.

The detailed procedure of the FDTD simulations has been explained elsewhere [7,9]. In brief, a short pulse is allowed to propagate, starting from an inner position to the microcavity, for 4 × 10^5^ time steps of 10^−17^ s each. Its Poynting vector is integrated in a plane that covers the cross section of the wire in the middle of the microcavity, as shown in Figure 5a. As the pulse is bounced off the two DBRs, it is propagated back and forth within the cavity resulting in the interference pattern that, after many reflections, yields the FDTD-simulated resonance pattern. 

Figure 5b shows normalized μ-PL spectra (solid lines) obtained by changing the excitation density of the laser spot so that the local temperature is 299 K (UV laser, OD 1 filter), 338 K (red laser, OD 0.3) and 360 K (red laser, no filter, full power), as calculated taking the maxima positions from Table 3. Simulated spectra (dashed lines and dot-dashed lines) at three different temperatures (RT, 338 K and 360 K) obtained with FDTD simulations are also shown. Dashed lines represent simulations using the anisotropic refractive index as obtained by ellipsometry in this work, while dot–dashed lines correspond to the same simulations using the anisotropic refractive index calculated from the data reported in ref. [11], but neglecting the contribution of the off-diagonal elements. A larger shift with temperature is observed for the experimental μ-PL spectra than that obtained with the FDTD simulations, i.e., simulations from the T-dependence of the refractive index obtained by ellipsometry underestimate the spectral shift when compared to that obtained from the measurement of the F-P resonances. This can also be expressed from the value of the thermo-optic coefficient as obtained from the previous works [9,10,11] and in this work, as shown in Table 3.

In spite of the results being similar to the previous works on the temperature-dependent refractive index of bulk β-Ga_2_O_3_ [10,11], the procedure to obtain the results presented in this work shows differences to those of other studies. The work by Bhaumik et al. used the prism method to obtain the refractive index at temperatures between room temperature (RT, 25 °C, i.e., 298 K) and 175 °C (448 K) [10]. They assumed that the reflected p and s components can be decoupled upon reflection. Although this is the case for isotropic materials, this is not the case for anisotropic materials, such as β-Ga_2_O_3_, in which the resulting reflection always shows a coupling of the p and s components. Moreover, their characterization only includes the refractive index along two crystal directions, the [010] and the direction perpendicular to the (100) planes. Although their approach might be a reasonable approximation, we believe that the use of generalized ellipsometry is a much better approach and should render more accurate data.

Regarding the work by Sturm et al. reported in [11], they used ellipsometry, and the analysis was similar to the one that we used. They had the advantage that they obtained the data with an additional orientation of the crystal, but had the disadvantage that they could not measure at multiple angles of incidence, only at 70° due to the use of a vacuum for their cryostat, which somehow limits the accuracy of their data. Note that we instead used three different angles of incidence, which means that for each crystal orientation, we have a factor three more data in different conditions for our fits. Nonetheless, the main difference between the work by Sturm et al. in [11] and the current work is the temperature range: they studied the variation in the refractive index at low temperatures—from 10 K to 300 K (room temperature)—while we report data in the 295 K to 595 K range. Even though the behaviour reported in [11] as a function of temperature could be extrapolated for higher temperatures, it is not straightforward that this would be a good approaching fact since it has already been reported that other properties of β-Ga_2_O_3_ do not show the same behaviour at temperatures below room temperature and at higher temperatures. For example, the electrical conductivity shows a much sharper change at temperatures below 300 K than above [20]. The trend is the same but the rate of variation is quite different.

In our previous work [9], the thermo-optic coefficient obtained from the F-P resonances was lower than that reported by the prism coupling technique in [10]. Therefore, while the trends as a function of temperature are the same, the value of this coefficient obtained with different experimental methods presents some variation. On the other hand, ellipsometry measurements, even though there is some uncertainty, are well-considered as a reliable way to assess the refractive index at RT for bulk materials. It is worth mentioning that heating in the ellipsometry experiments was conducted by placing the 2 mm thick crystal on a hot plate, while in the case of the F-P Ga_2_O_3_ cavities, the heating is localized at the laser spot region. Finally, it should also be pointed out that ellipsometry was performed in undoped material, while the nW-based cavities are Cr-doped. This might result in eventual changes in the refractive index due to Cr doping, as well as its behavior with temperature [21].

## 4. Conclusions

The anisotropic dispersion relations at different temperatures, *n_i_*(*λ*,*T*), in the 650–800 nm wavelength range are reported by ellipsometry in nominally undoped, bulk β-Ga_2_O_3_. The Mueller matrix formalism was used and good agreement with previous room temperature results was obtained. μ-PL spectra at different excitation laser powers from a Fabry–Perot microcavity built in a β-Ga_2_O_3_:Cr nanowire with DBRs were obtained. The spectral shift of the peaks allowed their local temperature at each excitation power to be calculated. Detailed FDTD simulations based on the obtained *n_i_*(*λ*,*T*) were carried out to assess such spectral shifts. The comparison of the FDTD simulations and the experimental results indicate an underestimation of the temperature dependence of the refractive index by ellipsometry as measured for the bulk crystal, and some other possible causes are discussed. The obtained thermo-optic coefficient is in the range of δ ≈ 10^−5^ K^−1^, although some variation in the values obtained by the different techniques is observed.

## Figures and Tables

**Table 1:** T-dependent dispersion relations derived from ellipsometry measurements for (100), (010) and (001) directions.

Coefficient	Quadratic Dependence with T	A	λ_i_ (μm)
ε_i,(100)_(T)	2.874 + 3.199 × 10^−6^ T + 3.732 × 10^−9^ T^2^	0.57	0.27
ε_i,(010)_(T)	3.046 + 4.416 × 10^−5^ T + 1.197 × 10^−9^ T^2^	0.57	0.27
ε_i,(001)_(T)	3.001 + 4.418 × 10^−5^ T + 7.600 × 10^−10^ T^2^	0.57	0.27
Temperature is in K.			

**Table 2:** Lorentzian fit parameters to PL curves excited with different powers, as shown in Figure 4d.

Peak #	T (K)	Center (nm)	Width (nm)
	299	732.207 ± 0.002	0.648 ± 0.008
3	338	732.6845 ± 0.0014	0.652 ± 0.007
	360	733.066 ± 0.002	0.688 ± 0.009
	299	741.404 ± 0.002	0.531 ± 0.011
4	338	741.880 ± 0.002	0.550 ± 0.010
	360	742.262 ± 0.002	0.592 ± 0.011

**Table 3:** Thermo-optic coefficient in β-Ga_2_O_3_ as calculated from results obtained in different works by several experimental methods.

δ (K^−1^)	Method	Temperature Range (K)	Reference
≈3.7 × 10^−5^	Prism coupling	298–448	[10]
≈1.8 × 10^−5^	Optical interferometry	150–400	[9]
≈6 × 10^−6^	Ellipsometry	10–300	[11]
≈5.7 × 10^−6^	Ellipsometry	295–595 K	This work

## Data Availability

The data that support the findings of this study are available from the corresponding author upon reasonable request.

## Supplementary Materials

The following supporting information can be downloaded at: https://www.mdpi.com/article/10.3390/nano13061126/s1, Figure S1: Geometry of the ellipsometry experimental setup and orientations; Figure S2: The measured experimental data are shown as points, the continuous lines are the fit to the data; Figure S3: Plots corresponding to measurements at 25 °C before and after heating up to 325 °C and the measurement at 325 °C for the Mueller matrix elements M12, M21, M33 and M34 at angle of incidence of 70º; Figure S4: (a) Evolution of Psi as a function of the temperature. (b) Evolution of average value of the refractive index during temperature variation. Figure S5: Fit with Lorentzian curves of the experimental PL peaks at three excitation powers for (a) peak #3 and (b) peak #4. References [9,15] are cited in the supplementary materials.
