# Supplementary material for: Temperature-Dependent Anisotropic Refractive Index in β-Ga2O3: Application in Interferometric Thermometers
Source: Nanomaterials (Basel). 2023 Mar 21;13(6):1126. doi: 10.3390/nano13061126 (PMC10058191; doi:10.3390/nano13061126)
Supplement: Supplementary file 1 [file nanomaterials-13-01126-s001.zip › nanomaterials-2272517-supplementary.pdf]

Supplementary materials

Temperature-Dependent Anisotropic Refractive Index in β-Ga_2O_3: Application in Interferometric Thermometers

Daniel Carrasco [1], Eva Nieto-Pinero [2], Manuel Alonso-Orts [1,3], Rosalía Serna [2], Jose M. San Juan [4], María L. Nó [4], Jani Jesenovec [5], John S. McCloy [5], Emilio Nogales [1,*] and Bianchi Méndez [1]

[1] Department Materials Physics, Faculty of Physics, Complutense University of Madrid, 28040 Madrid, Spain; daniecar@ucm.es (D.C.); manalo01@ucm.es (M.A.-O.); bianchi@fis.ucm.es (B.M.)
[2] Laser Processing Group, Instituto de Óptica (IO, CSIC), Serrano 121, 28006 Madrid, Spain; eva.nieto@csic.es (E.N.-P.); rosalia.serna@csic.es (R.S.)
[3] Institute of Solid State Physics, University of Bremen, Otto-Hahn-Allee 1, 28359 Bremen, Germany
[4] Department de Física, Facultad de Ciencia y Tecnología, Universidad del País Vasco UPV/EHU, Apdo. 644, 48080 Bilbao, Spain; jose.sanjuan@ehu.eus (J.M.S.J.); maria.no@ehu.es (M.L.N.)
[5] Crystals and Semiconductors Group, Institute of Materials Research, Washington State University, Pullman, WA 99164, USA; jani.jesenovec@baesystems.com (J.J.); john.mccloy@wsu.edu (J.S.M.)
* Correspondence: enogales@ucm.es

Table of contents

I. **Ellipsometry analysis**
II. **Simulations with OptiFDTD**

I. Ellipsometry

Figure S1 shows the defined geometry for the ellipsometry experiments.

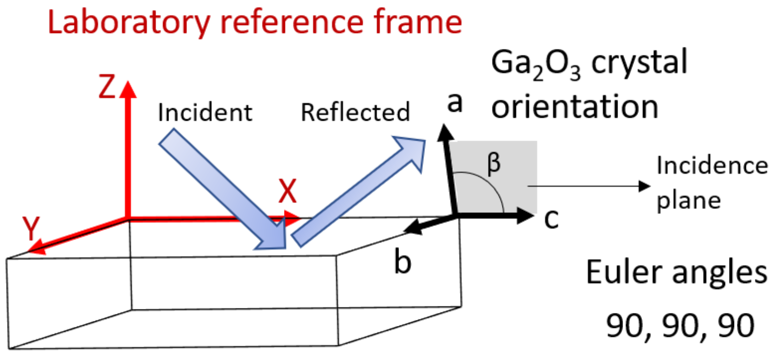

Figure S1. Geometry of the ellipsometry experimental setup and orientations.

At 25ºC (room temperature) we have used as starting data for the fits those provided by Sturm [1] in the visible range, where there is no absorption.

Figure S2 shows the quality of the fit for two Mueller matrix elements (M12 and M33) for all the incidence angles and for the orientation corresponding to Euler angles (90,90,90)

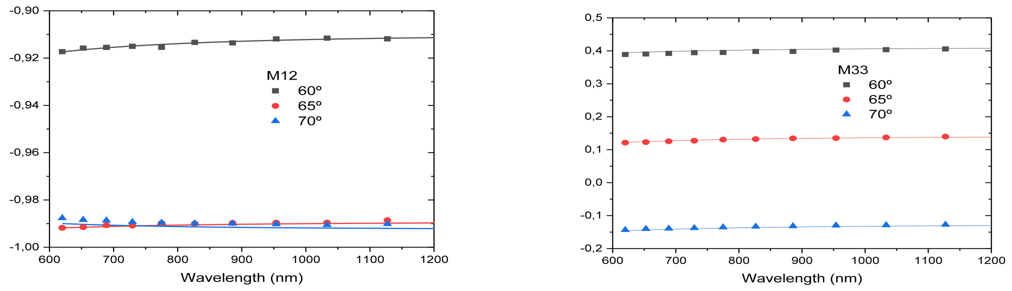

Figure S2. The measured experimental data are show as points, the continuous lines are the fit to the data.

The measurements show a clear change when the temperature is increased up to 325 ºC. Figure S3 shows Mueller matrix elements with non-zero values: M12, M21, M33 and M34. They clearly decrease at 325ºC as compared to 25ºC. These example plots correspond to measurements at Euler angles 90, 90, 90, and at incidence angle of 70º. Note that two measurements at 25ºC are shown: before and after heating at 325ºC. They show very good agreement.

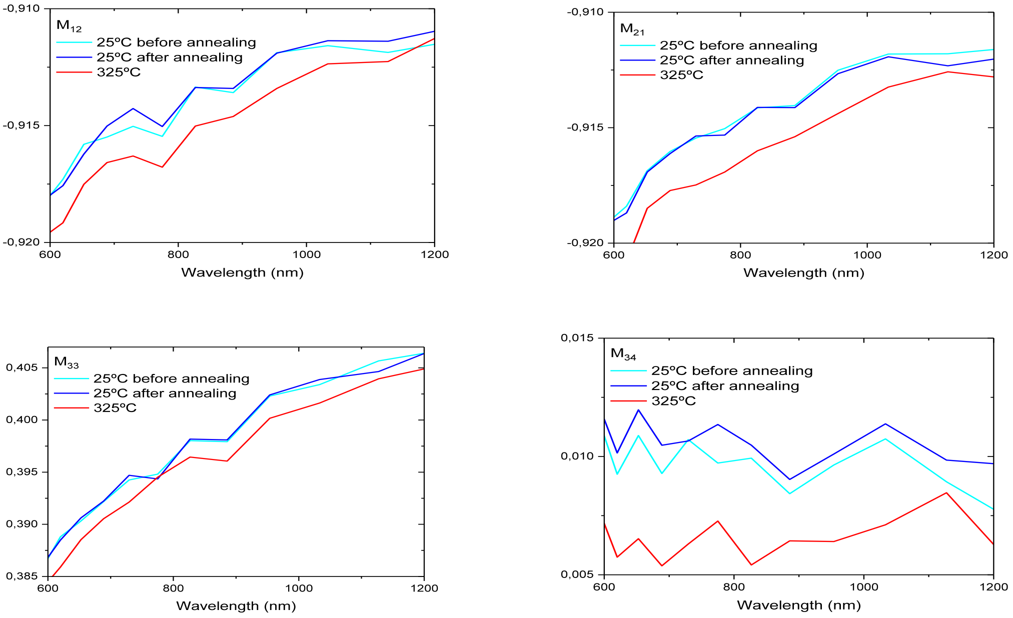

Figure S3. Plots corresponding to measurements at 25ºC before and after heating up to 325ºC, and the measurement at 325 ºC for the Mueller matrix elements M12, M21, M33 and M34 at angle of incidence of 70º.

In order to observe the evolution of the ellipsometric parameters during the heating cycle we have acquired angle Psi at the angle of incidence of 65º for a few wavelengths in the range from 600 to 800 nm. The heating rate was 2 K/min. Figure S4(a) shows the evolution of Psi as a function of the temperature. We observe a linear decrease of the Psi value, associated to a linear increase of the refractive index values. This results in the variation of the average refractive index shown in figure S4(b).

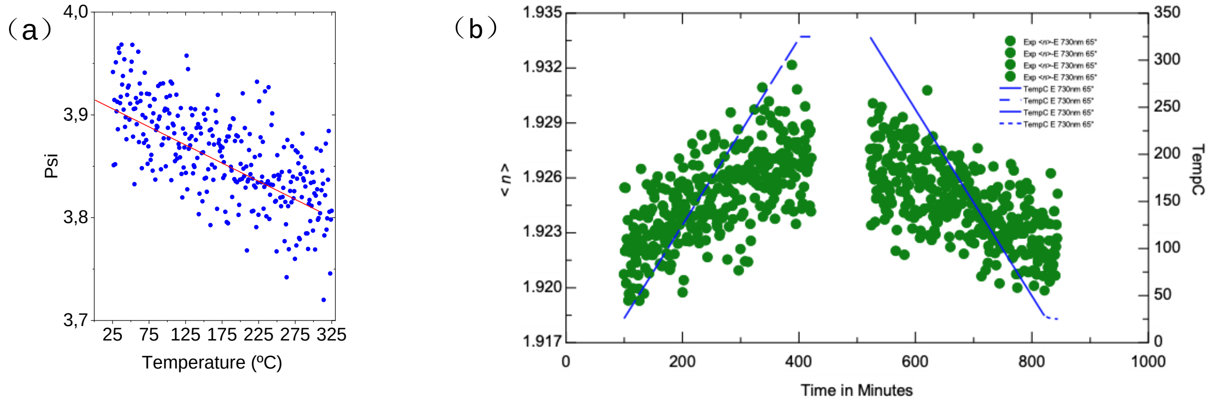

Figure S4. (a) Evolution of Psi as a function of the temperature. (b) Evolution of average value of the refractive index during temperature variation.

II. Experimental PL peaks

Peak position vs. temperature calibration curves

The calibration curves for the peaks were obtained for this sensing aim [2]

$$\lambda_{\#2}(T) = \lambda_{\#2}(295\ K) + 1{,}15 \cdot 10^{-5} T^2 + 5{,}5 \cdot 10^{-3} T - 2{,}65 \qquad \text{(S1.a)}$$

$$\lambda_{\#3}(T) = \lambda_{\#3}(295\ K) + 1{,}14 \cdot 10^{-5} T^2 + 6{,}1 \cdot 10^{-3} T - 2{,}8 \qquad \text{(S1.b)}$$

$$\lambda_{\#4}(T) = \lambda_{\#4}(295\ K) + 1{,}19 \cdot 10^{-5} T^2 + 6{,}2 \cdot 10^{-3} T - 2{,}91 \qquad \text{(S1.c)}$$

where λ is expressed in nm and T in K.

The position of the experimental F-P peaks obtained by PL has been obtained by Lorentzian fits, as shown in figure S5.

(a)

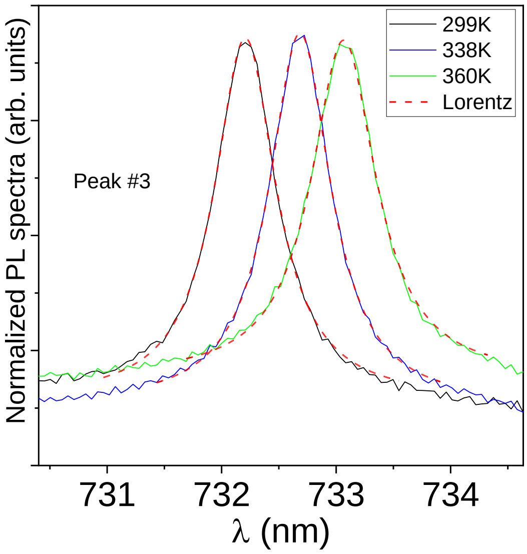

Model	Lorentz
Equation	y = y0 + (2*A/pi)*(w/(4*(x-xc)^2 + w^2))
Plot	Normalized1
y0	0,31281 ± 0,00241
xc	732,20716 ± 0,00171
w	0,64825 ± 0,0077
A	0,64198 ± 0,00795
Reduced Chi-Sqr	5,45013E-5
R-Square (COD)	0,99852
Adj. R-Square	0,99842

Model	Lorentz
Equation	y = y0 + (2*A/pi)*(w/(4*(x-xc)^2 + w^2))
Plot	Normalized1
y0	0,30294 ± 0,0022
xc	732,68456 ± 0,00143
w	0,65154 ± 0,00659
A	0,6641 ± 0,00712
Reduced Chi-Sqr	3,99262E-5
R-Square (COD)	0,99898
Adj. R-Square	0,99892

Model	Lorentz
Equation	y = y0 + (2*A/pi)*(w/(4*(x-xc)^2 + w^2))
Plot	Normalized1
y0	0,3512 ± 0,00267
xc	733,0658 ± 0,00203
w	0,68795 ± 0,00936
A	0,63596 ± 0,00914
Reduced Chi-Sqr	6,32325E-5
R-Square (COD)	0,99803
Adj. R-Square	0,99791

(b)

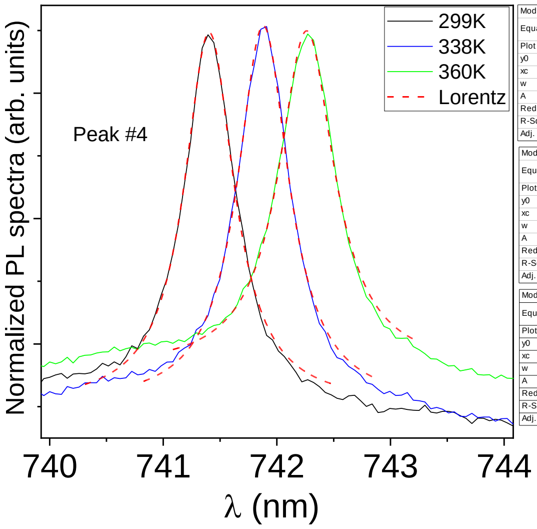

Model	Lorentz
Equation	y = y0 + (2*A/pi)*(w/(4*(x-xc)^2 + w^2))
Plot	Normalized1
y0	0,30251 ± 0,00387
xc	741,40416 ± 0,00247
w	0,53108 ± 0,01098
A	0,49582 ± 0,01058
Reduced Chi-Sqr	1,23751E-4
R-Square (COD)	0,99626
Adj. R-Square	0,99598

Model	Lorentz
Equation	y = y0 + (2*A/pi)*(w/(4*(x-xc)^2 + w^2))
Plot	Normalized1
y0	0,30166 ± 0,00375
xc	741,88025 ± 0,0021
w	0,55054 ± 0,00988
A	0,52405 ± 0,01009
Reduced Chi-Sqr	8,92237E-5
R-Square (COD)	0,99746
Adj. R-Square	0,99725

Model	Lorentz
Equation	y = y0 + (2*A/pi)*(w/(4*(x-xc)^2 + w^2))
Plot	Normalized1
y0	0,35887 ± 0,00366
xc	742,26194 ± 0,00241
w	0,59187 ± 0,01142
A	0,50256 ± 0,01048
Reduced Chi-Sqr	8,73161E-5
R-Square (COD)	0,99688
Adj. R-Square	0,99663

Figure S5. Fit with Lorentzian curves of the experimental PL peaks at three excitation powers for (a) peak #3 and (b) peak #4.

REFERENCES

[1] C. Sturm, J. Furthmüller, F. Bechstedt, R. Schmidt-Grund, and M. Grundmann. Dielectric tensor of monoclinic Ga_2O_3 single crystals in the spectral range 0.5-8.5 eV, *APL Materials* **2017**, 3, 106106. https://doi.org/10.1063/1.4934705

[2] Manuel Alonso-Orts, Daniel Carrasco, José M. San Juan, María Luisa Nó, Alicia de Andrés, Emilio Nogales, and Bianchi Méndez, Wide dynamic range thermometer based on luminescent optical cavities in Ga_2O_3:Cr nanowires. *Small* **2022**, 18, 2105355. DOI: 10.1002/smll.202105355
